# Critical iron deficiency anemia with record low hemoglobin: a case report

**DOI:** 10.1186/s13256-021-03024-9

**Published:** 2021-09-13

**Authors:** Audrey L. Chai, Owen Y. Huang, Rastko Rakočević, Peter Chung

**Affiliations:** 1 Department of Medicine, University of Southern California, Los Angeles, CA USA; 2 Division of Pulmonary, Critical Care and Sleep Medicine, Department of Medicine, University of Southern California, Los Angeles, CA USA

**Keywords:** Anemia, Menometrorrhagia, Iron deficiency, Critical care, Transfusion

## Abstract

**Background:**

Anemia is a serious global health problem that affects individuals of all ages but particularly women of reproductive age. Iron deficiency anemia is one of the most common causes of anemia seen in women, with menstruation being one of the leading causes. Excessive, prolonged, and irregular uterine bleeding, also known as menometrorrhagia, can lead to severe anemia. In this case report, we present a case of a premenopausal woman with menometrorrhagia leading to severe iron deficiency anemia with record low hemoglobin.

**Case presentation:**

A 42-year-old Hispanic woman with no known past medical history presented with a chief complaint of increasing fatigue and dizziness for 2 weeks. Initial vitals revealed temperature of 36.1 °C, blood pressure 107/47 mmHg, heart rate 87 beats/minute, respiratory rate 17 breaths/minute, and oxygen saturation 100% on room air. She was fully alert and oriented without any neurological deficits. Physical examination was otherwise notable for findings typical of anemia, including: marked pallor with pale mucous membranes and conjunctiva, a systolic flow murmur, and koilonychia of her fingernails. Her initial laboratory results showed a critically low hemoglobin of 1.4 g/dL and severe iron deficiency. After further diagnostic workup, her profound anemia was likely attributed to a long history of menometrorrhagia, and her remarkably stable presentation was due to impressive, years-long compensation. Over the course of her hospital stay, she received blood transfusions and intravenous iron repletion. Her symptoms of fatigue and dizziness resolved by the end of her hospital course, and she returned to her baseline ambulatory and activity level upon discharge.

**Conclusions:**

Critically low hemoglobin levels are typically associated with significant symptoms, physical examination findings, and hemodynamic instability. To our knowledge, this is the lowest recorded hemoglobin in a hemodynamically stable patient not requiring cardiac or supplemental oxygen support.

## Background

Anemia and menometrorrhagia are common and co-occurring conditions in women of premenopausal age [1, 2]. Analysis of the global anemia burden from 1990 to 2010 revealed that the prevalence of iron deficiency anemia, although declining every year, remained significantly high, affecting almost one in every five women [1]. Menstruation is considered largely responsible for the depletion of body iron stores in premenopausal women, and it has been estimated that the proportion of menstruating women in the USA who have minimal-to-absent iron reserves ranges from 20% to 65% [3]. Studies have quantified that a premenopausal woman’s iron storage levels could be approximately two to three times lower than those in a woman 10 years post-menopause [4]. Excessive and prolonged uterine bleeding that occurs at irregular and frequent intervals (menometrorrhagia) can be seen in almost a quarter of women who are 40–50 years old [2]. Women with menometrorrhagia usually bleed more than 80 mL, or 3 ounces, during a menstrual cycle and are therefore at greater risk for developing iron deficiency and iron deficiency anemia. Here, we report an unusual case of a 42-year-old woman with a long history of menometrorrhagia who presented with severe anemia and was found to have a record low hemoglobin level.

## Case presentation

A 42-year-old Hispanic woman with no known past medical history presented to our emergency department with the chief complaint of increasing fatigue and dizziness for 2 weeks and mechanical fall at home on day of presentation.

**Fig. 1:**
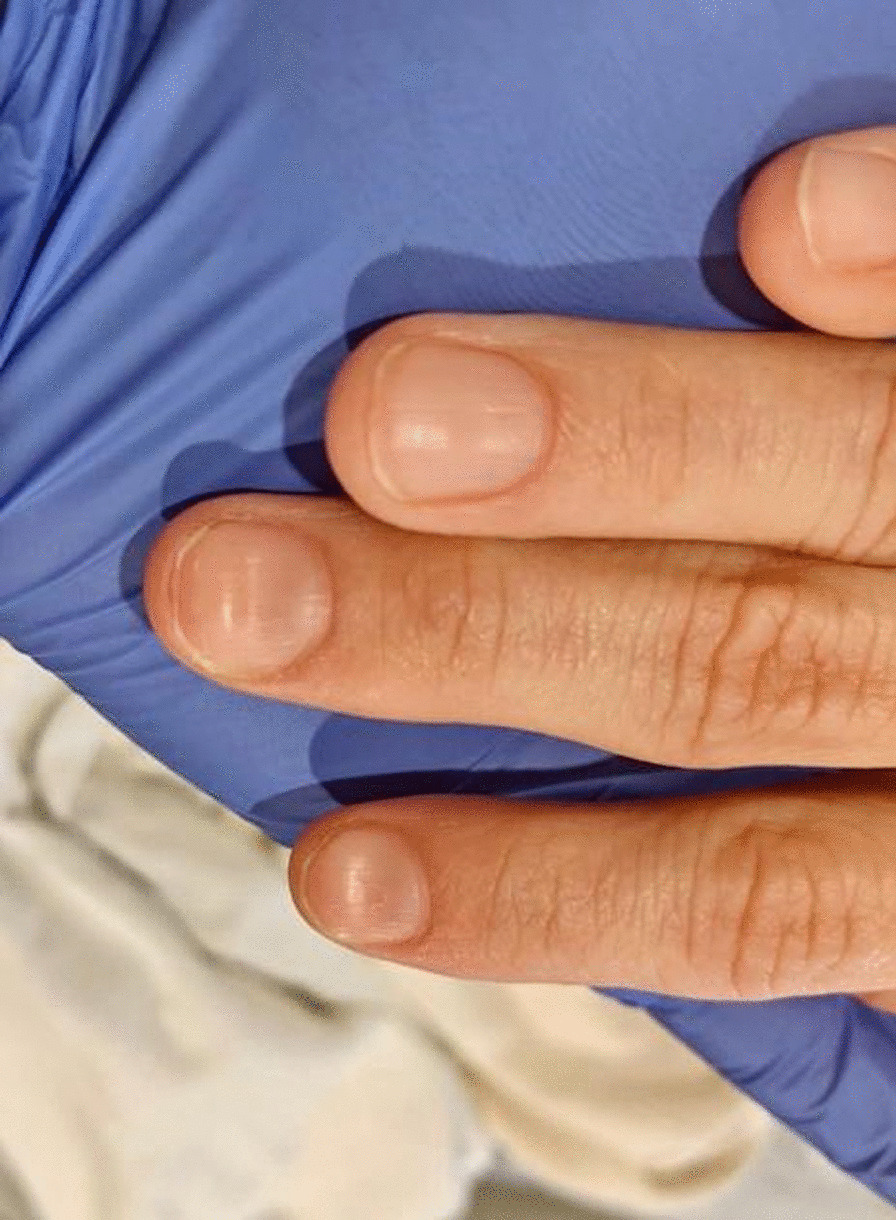
Koilonychia, as seen in our patient above, is a nail disease commonly seen in hypochromic anemia, especially iron deficiency anemia, and refers to abnormally thin nails that have lost their convexity, becoming flat and sometimes concave in shape

On physical examination, she was afebrile (36.1 °C), blood pressure was 107/47 mmHg with a mean arterial pressure of 69 mmHg, heart rate was 87 beats per minute (bpm), respiratory rate was 17 breaths per minute, and oxygen saturation was 100% on room air. Her height was 143 cm and weight was 45 kg (body mass index 22). She was fully alert and oriented to person, place, time, and situation without any neurological deficits and was speaking in clear, full sentences. She had marked pallor with pale mucous membranes and conjunctiva. She had no palpable lymphadenopathy. She was breathing comfortably on room air and displayed no signs of shortness of breath. Her cardiac examination was notable for a grade 2 systolic flow murmur. Her abdominal examination was unremarkable without palpable masses. On musculoskeletal examination, her extremities were thin, and her fingernails demonstrated koilonychia (Fig. 1). She had full strength in lower and upper extremities bilaterally, even though she required assistance with ambulation secondary to weakness and used a wheelchair for mobility for 2 weeks prior to admission. She declined a pelvic examination. No bleeding was noted in any part of her physical examination.

She was admitted directly to the intensive care unit after her hemoglobin was found to be critically low at 1.4 g/dL on two consecutive measurements with an unclear etiology of blood loss at the time of presentation. Note that no intravenous fluids were administered prior to obtaining the hemoglobin levels. Upon collecting further history from the patient, she revealed that she has had a lifetime history of extremely heavy menstrual periods: Since menarche at the age of 10 years when her periods started, she has been having irregular menstruation, with periods occurring every 2–3 weeks, sometimes more often. She bled heavily for the entire 5–7 day duration of her periods; she quantified soaking at least seven heavy flow pads each day with bright red blood as well as large-sized blood clots. Since the age of 30 years, her periods had also become increasingly heavier, with intermittent bleeding in between cycles, stating that lately she bled for “half of the month.” She denied any other sources of bleeding.

**Fig. 2:**
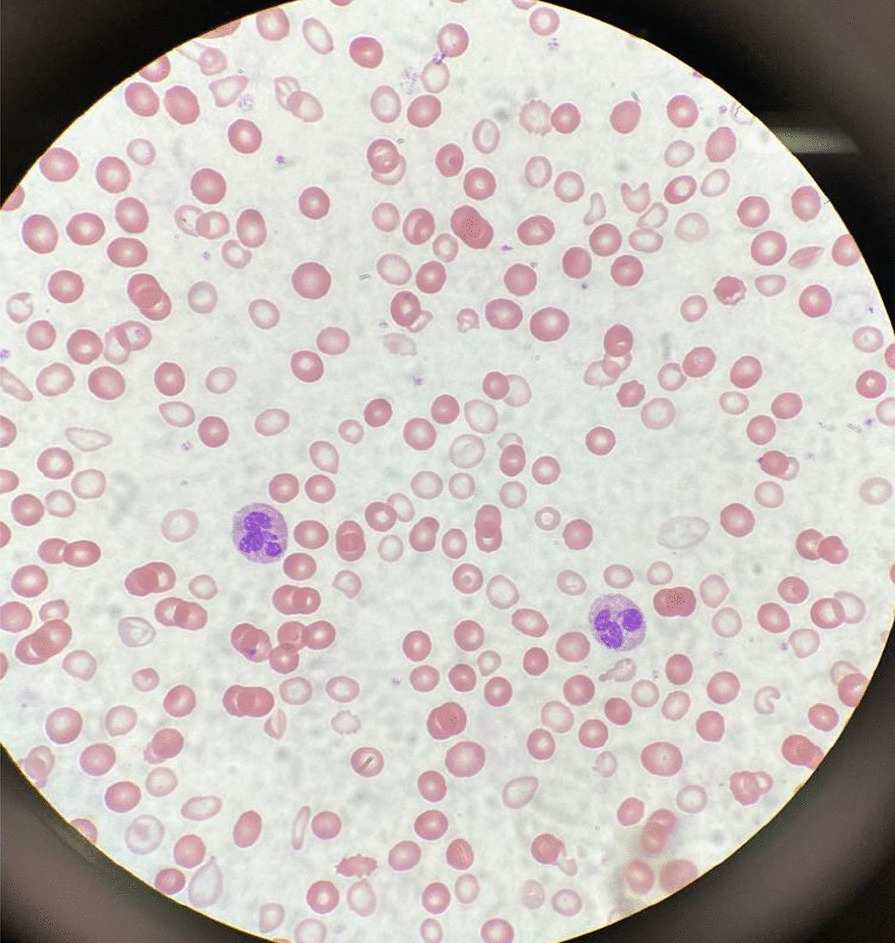
A peripheral smear from the patient after receiving one packed red blood cell transfusion is shown. Small microcytic red blood cells are seen, many of which are hypochromic and have a large zone of pallor with a thin pink peripheral rim. A few characteristic poikilocytes (small elongated red cells also known as pencil cells) are also seen in addition to normal red blood cells (RBCs) likely from transfusion

Initial laboratory data are summarized in Table 1. Her hemoglobin (Hgb) level was critically low at 1.4 g/dL on arrival, with a low mean corpuscular volume (MCV) of < 50.0 fL. Hematocrit was also critically low at 5.8%. Red blood cell distribution width (RDW) was elevated to 34.5%, and absolute reticulocyte count was elevated to 31 × 10^9^/L. Iron panel results were consistent with iron deficiency anemia, showing a low serum iron level of 9 μg/dL, elevated total iron-binding capacity (TIBC) of 441 μg/dL, low Fe Sat of 2%, and low ferritin of 4 ng/mL. Vitamin B12, folate, hemolysis labs [lactate dehydrogenase (LDH), haptoglobin, bilirubin], and disseminated intravascular coagulation (DIC) labs [prothrombin time (PT), partial thromboplastin time (PTT), fibrinogen, d-dimer] were all unremarkable. Platelet count was 232,000/mm^3^. Peripheral smear showed erythrocytes with marked microcytosis, anisocytosis, and hypochromia (Fig. 2). Of note, the patient did have a positive indirect antiglobulin test (IAT); however, she denied any history of pregnancy, prior transfusions, intravenous drug use, or intravenous immunoglobulin (IVIG). Her direct antiglobulin test (DAT) was negative.

**Table 1:** Summary o﻿f the patient’s laboratory data

Laboratory	On admission	Reference range
WBC	5400/mm^3^	4500–10,000/mm^3^
RBC	1.14 M/mm^3^	3.9–5.1 M/mm^3^
Hgb	1.4 g/dL	12.0–14.6 g/dL
Hct	5.8%	36.0–44.0%
MCV	< 50.0 fL	82.0–97.0 fL
RDW	21.9%	12–15%
Platelet	232,000/mm^3^	160,000–360,000/mm^3^
Retic Pct	2.7%	0.6–2.4%
Abs retic	31 × 10^9^/L	29–116 × 10^9^/L
PT	17.6 seconds	11.8–14.4 seconds
INR	1.46	0.87–1.13
PTT	32.5 seconds	24.4–36.6 seconds
Fibrinogen	332 mg/dL	237–481 mg/dL
d-dimer	2.16 μg/mL	≤ 0.49 μg/mL
Alk Phos	150 U/L	35–104 U/L
AST	81 U/L	10–35 U/L
ALT	36 U/L	10–35 U/L
Protein total	5.6 g/dL	6–8 g/dL
Bili total	0.5 mg/dL	≤ 1.0 mg/dL
Bili direct	0.3 mg/dL	≤ 0.3 mg/dL
Iron	9 μg/dL	37–145 μg/dL
TIBC	441 μg/dL	250–430 μg/dL
Iron sat	2%	15–50%
Ferritin	4 ng/mL	12–263 ng/mL
Haptoglobin	168 mg/dL	30–200 mg/dL
LDH	206 U/L	135–225 U/L
Folate	12.8 ng/mL	≥ 4.6 ng/mL
Vitamin B12	1,019 pg/mL	232–1245 pg/mL

*WBC* White Blood Cell Count, *RBC* Red Blood Cell Count, *Hgb* Hemoglobin, *Hct* Hematocrit, *MCV* Mean Corpuscular Volume, *RDW* Red Cell Distribution Width, *Retic Pct* Reticulocyte Percentage, *Abs retic* Absolute Reticulocyte Count, *PT* Prothrombin Time, *INR* International Normalized Ratio, *PTT* Partial Thromboplastin Time, *Alk Phos* Alkaline Phosphatase, *AST* Aspartate transaminase, *ALT* Alanine Aminotransferase, *Bili total* Bilirubin total, *Bili direct* Direct bilirubin, *TIBC* Total iron binding capacity, *LDH* Lactate Dehydrogenase

**Fig. 3:**
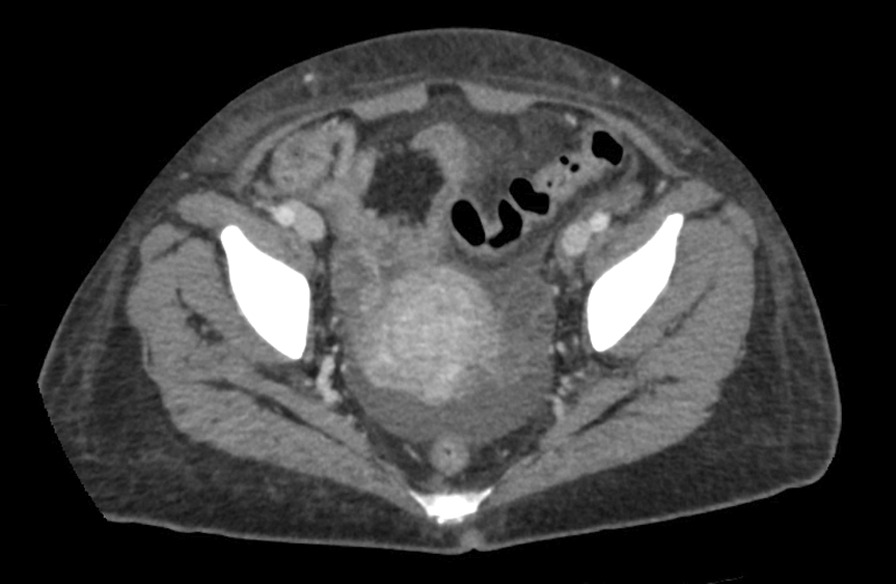
Computed tomography (CT) of the abdomen and pelvis with contrast was obtained revealing an approximately 3.5 × 3.0 cm heterogeneously enhancing mass protruding into the endometrial canal favored to represent an intracavitary submucosal leiomyoma

A transvaginal ultrasound and endometrial biopsy were offered, but the patient declined. Instead, a computed tomography (CT) abdomen and pelvis with contrast was performed, which showed a 3.5-cm mass protruding into the endometrium, favored to represent an intracavitary submucosal leiomyoma (Fig. 3). Aside from her abnormal uterine bleeding (AUB), the patient was without any other significant personal history, family history, or lab abnormalities to explain her severe anemia.

The patient’s presenting symptoms of fatigue and dizziness are common and nonspecific symptoms with a wide range of etiologies. Based on her physical presentation—overall well-appearing nature with normal vital signs—as well as the duration of her symptoms, we focused our investigation on chronic subacute causes of fatigue and dizziness rather than acute medical causes. We initially considered a range of chronic medical conditions from cardiopulmonary to endocrinologic, metabolic, malignancy, rheumatologic, and neurological conditions, especially given her reported history of fall. However, once the patient’s lab work revealed a significantly abnormal complete blood count and iron panel, the direction of our workup shifted towards evaluating hematologic causes.

With such a critically low Hgb on presentation (1.4 g/dL), we evaluated for potential sources of blood loss and wanted to first rule out emergent, dangerous causes: the patient’s physical examination and reported history did not elicit any concern for traumatic hemorrhage or common gastrointestinal bleeding. She denied recent or current pregnancy. Her CT scan of abdomen and pelvis was unremarkable for any pathology other than a uterine fibroid. The microcytic nature of her anemia pointed away from nutritional deficiencies, and she lacked any other medical comorbidities such as alcohol use disorder, liver disease, or history of substance use. There was also no personal or family history of autoimmune disorders, and the patient denied any history of gastrointestinal or extraintestinal signs and/or symptoms concerning for absorptive disorders such as celiac disease. We also eliminated hemolytic causes of anemia as hemolysis labs were all normal. We considered the possibility of inherited or acquired bleeding disorders, but the patient denied any prior signs or symptoms of bleeding diatheses in her or her family. The patient’s reported history of menometrorrhagia led to the likely cause of her significant microcytic anemia as chronic blood loss from menstruation leading to iron deficiency.

Over the course of her 4-day hospital stay, she was transfused 5 units of packed red blood cells and received 2 g of intravenous iron dextran. Hematology and Gynecology were consulted, and the patient was administered a medroxyprogesterone (150 mg) intramuscular injection on hospital day 2. On hospital day 4, she was discharged home with follow-up plans. Her hemoglobin and hematocrit on discharge were 8.1 g/dL and 24.3%, respectively. Her symptoms of fatigue and dizziness had resolved, and she was back to her normal baseline ambulatory and activity level.

## Discussion and conclusions

This patient presented with all the classic signs and symptoms of iron deficiency: anemia, fatigue, pallor, koilonychia, and labs revealing marked iron deficiency, microcytosis, elevated RDW, and low hemoglobin. To the best of our knowledge, this is the lowest recorded hemoglobin in an awake and alert patient breathing ambient air. There have been previous reports describing patients with critically low Hgb levels of < 2 g/dL: A case of a 21-year old woman with a history of long-lasting menorrhagia who presented with a Hgb of 1.7 g/dL was reported in 2013 [5]. This woman, although younger than our patient, was more hemodynamically unstable with a heart rate (HR) of 125 beats per minute. Her menorrhagia was also shorter lasting and presumably of larger volume, leading to this hemoglobin level. It is likely that her physiological regulatory mechanisms did not have a chance to fully compensate. A 29-year-old woman with celiac disease and bulimia nervosa was found to have a Hgb of 1.7 g/dL: she presented more dramatically with severe fatigue, abdominal pain and inability to stand or ambulate. She had a body mass index (BMI) of 15 along with other vitamin and micronutrient deficiencies, leading to a mixed picture of iron deficiency and non-iron deficiency anemia [6]. Both of these cases were of reproductive-age females; however, our patient was notably older (age difference of > 20 years) and had a longer period for physiologic adjustment and compensation.

Lower hemoglobin, though in the intraoperative setting, has also been reported in two cases—a patient undergoing cadaveric liver transplantation who suffered massive bleeding with associated hemodilution leading to a Hgb of 0.6 g/dL [7] and a patient with hemorrhagic shock and extreme hemodilution secondary to multiple stab wounds leading to a Hgb of 0.7 g/dL [8]. Both patients were hemodynamically unstable requiring inotropic and vasopressor support, had higher preoperative hemoglobin, and were resuscitated with large volumes of colloids and crystalloids leading to significant hemodilution. Both were intubated and received 100% supplemental oxygen, increasing both hemoglobin-bound and dissolved oxygen. Furthermore, it should be emphasized that the deep anesthesia and decreased body temperature in both these patients minimized oxygen consumption and increased the available oxygen in arterial blood [9].

Our case is remarkably unique with the lowest recorded hemoglobin not requiring cardiac or supplemental oxygen support. The patient was hemodynamically stable with a critically low hemoglobin likely due to chronic, decades-long iron deficiency anemia of blood loss. Confirmatory workup in the outpatient setting is ongoing. The degree of compensation our patient had undergone is impressive as she reported living a very active lifestyle prior to the onset of her symptoms (2 weeks prior to presentation), she routinely biked to work every day, and maintained a high level of daily physical activity without issue.

In addition, while the first priority during our patient’s hospital stay was treating her severe anemia, her education became an equally important component of her treatment plan. Our institution is the county hospital for the most populous county in the USA and serves as a safety-net hospital for many vulnerable populations, most of whom have low health literacy and a lack of awareness of when to seek care. This patient had been experiencing irregular menstrual periods for more than three decades and never sought care for her heavy bleeding. She, in fact, had not seen a primary care doctor for many years nor visited a gynecologist before. We emphasized the importance of close follow-up, self-monitoring of her symptoms, and risks with continued heavy bleeding. It is important to note that, despite the compensatory mechanisms, complications of chronic anemia left untreated are not minor and can negatively impact cardiovascular function, cause worsening of chronic conditions, and eventually lead to the development of multiorgan failure and even death [10, 11].

## Data Availability

All data generated or analyzed during this study are included in this published article.
